# The application of virtual reality exposure versus relaxation training in music performance anxiety: a randomized controlled study

**DOI:** 10.1186/s12888-023-05040-z

**Published:** 2023-08-01

**Authors:** Daniel Bellinger, Kristin Wehrmann, Anna Rohde, Maria Schuppert, Stefan Störk, Michael Flohr-Jost, Dominik Gall, Paul Pauli, Jürgen Deckert, Martin J. Herrmann, Angelika Erhardt-Lehmann

**Affiliations:** 1 Department of Psychiatry, Psychosomatics, and Psychotherapy, Center for Mental Health, University Hospital Würzburg, Würzburg, Germany; 2 University of Music Würzburg, Würzburg, Germany; 3 Department Clinical Research & Epidemiology, Comprehensive Heart Failure Center Würzburg, University Hospital Würzburg, Würzburg, Germany; 4 Department of Psychology (Biological Psychology, Clinical Psychology, and Psychotherapy), Center for Mental Health, University of Würzburg, Würzburg, Germany; 5 Department of Translational Research in Psychiatry, Max Planck Institute of Psychiatry, Munich, Germany

**Keywords:** Music performance anxiety, Virtual reality exposure therapy, Progressive muscle relaxation, Heart rate variability

## Abstract

**Background:**

Performance anxiety is the most frequently reported anxiety disorder among professional musicians. Typical symptoms are - on a physical level - the consequences of an increase in sympathetic tone with cardiac stress, such as acceleration of heartbeat, increase in blood pressure, increased respiratory rate and tremor up to nausea or flush reactions. These symptoms can cause emotional distress, a reduced musical and artistical performance up to an impaired functioning. While anxiety disorders are preferably treated using cognitive-behavioral therapy with exposure, this approach is rather difficult for treating music performance anxiety since the presence of a public or professional jury is required and not easily available. The use of virtual reality (VR) could therefore display an alternative. So far, no therapy studies on music performance anxiety applying virtual reality exposure therapy have investigated the therapy outcome including cardiovascular changes as outcome parameters.

**Methods:**

This mono-center, prospective, randomized and controlled clinical trial has a pre-post design with a follow-up period of 6 months. 46 professional and semi-professional musicians will be recruited and allocated randomly to an VR exposure group or a control group receiving progressive muscle relaxation training. Both groups will be treated over 4 single sessions. Music performance anxiety will be diagnosed based on a clinical interview using ICD-10 and DSM-5 criteria for specific phobia or social anxiety. A behavioral assessment test is conducted three times (pre, post, follow-up) in VR through an audition in a concert hall. Primary outcomes are the changes in music performance anxiety measured by the German *Bühnenangstfragebogen* and the cardiovascular reactivity reflected by heart rate variability (HRV). Secondary outcomes are changes in blood pressure, stress parameters such as cortisol in the blood and saliva, neuropeptides, and DNA-methylation.

**Discussion:**

The trial investigates the effect of VR exposure in musicians with performance anxiety compared to a relaxation technique on anxiety symptoms and corresponding cardiovascular parameters. We expect a reduction of anxiety but also a consecutive improvement of HRV with cardiovascular protective effects.

**Trial registration:**

: This study was registered on clinicaltrials.gov. (ClinicalTrials.gov Number: NCT05735860)

## Background

### Definition, prevalence and treatment of MPA: current state of research

**Fig. 1:**
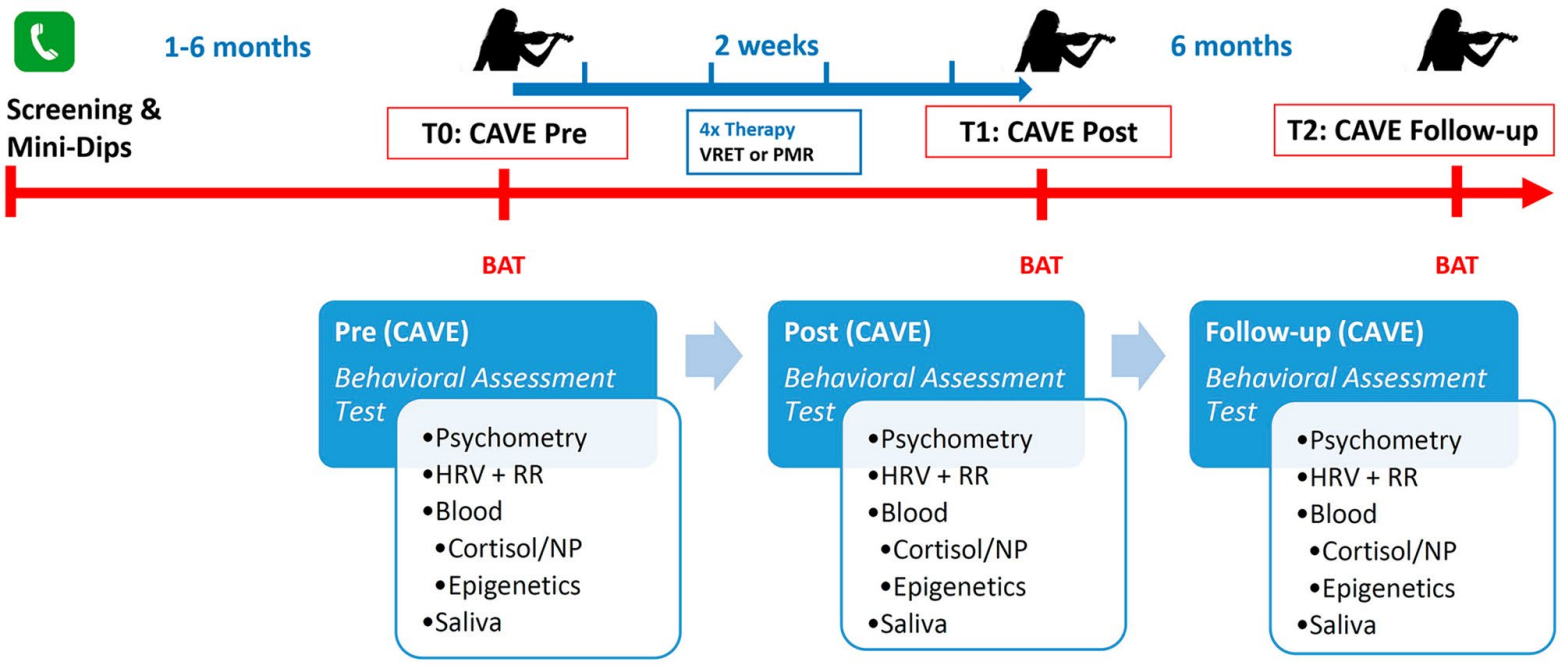
Timeline of screening and assessment at time points T0, T1, and T2 during the study

Music performance anxiety (MPA) can be defined as a subentity (“performance only” subtype) of social anxiety according to DSM-5 (F40.10) or as specific phobia as coded by the ICD-10 (F40.2)[1]. Although a common definition of the disorder ist still missing, it has to be differentiated from normal stage fright [1, 2]. According to the definition by Dianne Kenny, one of the leading researchers in the field of MPA, MPA is a “*combination of affective, cognitive, somatic and behavioural symptoms which may occur in a range of performance settings, but is usually more severe in settings involving high ego investment and evaluative threat*” [3, p. 433]. Whereas stage fright is a common and normal phenomenon among professional musicians, usually considered positive and performance-enhancing, MPA is considered a pathological fear that significantly affects the musical and artistic performance of the individual and therefore causes psychological distress and/or impaired functioning. In individual cases, severe MPA can even lead to the termination of the professional career. Since the transition from stage fright to MPA is smooth, the discrimination of both concepts is not always easy [2]. Mumm et al. draw an analogy to the *Yerkes-Dodson* curve to describe the dependence of arousal level on performance quality. An optimal condition of the arousal enables an optimal performance quality which is in the form of a U-shaped curve. An arousal level that is too high, as occurs with MPA, has a correspondingly negative effect on performance quality [2] (Fig. 1). Fernholz at al. [1] state MPA as the most frequently reported (anxiety) disorder among professional musicians. The prevalence of MPA varies in studies between 17% and 59% [1, 2] which can be attributed to the heterogeneity of sample size, methodology and definitions of MPA in these studies. Considering a minimum standard of criteria-based and standardized diagnostic, a prevalence of 15–25% can be assumed of which again only a small percentage of approximately 15% seems to seek professional help [1, 2, 4]. In a recent study based on questionnaires of 532 musicians Spahn et al. characterize three different types of MPA depending on symptoms, functional coping and performance-related self-efficacy [5]. Typical symptoms of performance-reducing MPA include subjectively experienced fear of failure or loss of control and on a physical level the consequences of an increase in sympathetic activity with cardiac stress. These include an increase in heart rate (tachycardia), blood pressure as well as an increased respiratory rate, tremor, nausea and flush reactions in the face. With regard to control of breathing, attachment, voice, and hand functions, these symptoms can seriously impact the sensorimotor performance, but also the flow state of musicians during music performance [6].

Cardiac stress with tachycardias and a mean heart rate of 130–160 bpm are very common in solo performances by instrumentalists [7, 8]. This aligns with observations in high-performance athletes, which are known to have a higher risk to experience cardiac arrhythmias [9–11]. In extreme cases, musicians of different musical genres may even suffer an acute cardiovascular event during a performance, such as a heart attack (Mariss Jansons 1996 (1943–2019)) or cerebral hemorrhage (Simon Barere, 1896–1951; Giuseppe Sinopoli, 1946–2001). Due to the concomitant sympathetic activation, musicians with MPA often resort to beta-blockers, although the reduction in physical signs and symptoms does not guarantee the reduction of psycho-emotional and cognitive factors (e.g. dysfunctional thoughts and misgivings) [3]. In general, serious mental illnesses are associated with high cardiovascular disease risk [12]. Although cardiovascular symptoms have been investigated in MPA [7, 13, 14], there is no study so far that investigated the therapeutic effects on sympathetic activity and cardiovascular reactivity for musicians with MPA. Guyon et al. rightly point out that studies about the “psychobiological and performance-related concomitants of MPA are limited” [15]. Their recent study is based on a promising approach of the biopsychosocial model of challenge and threat and investigates changes in psychobiological responses in MPA – data which is long overdue in the field of MPA.

Furthermore, no studies have been performed on blood-based therapy-related biomarkers in MPA. However, blood is currently the medium that is available for diagnostic purposes with little expenditure of time and personnel, as well as not being burdensome for the patient. Some first evidence of the potential utility of such blood-based biomarkers for the prediction of exposure therapy response comes from metabolomic and epigenetic studies in patients with Panic Disorder [16–18].

With regard to therapy of MPA, there exist manifold coping strategies and therapeutic interventions in the field of evidence based medicine such as cognitive behavioral therapy (CBT), medication with beta blockers but also complementary therapy methods like music therapy or Alexander technique described in detail by Fernholz et al. [1]. Early studies with behavioral interventions have been done since the 1990s. One of these initial studies by Clark & Agras [19] examined 94 musicians in a double-blind, randomized, placebo-controlled study. The anxiolytic buspirone was compared with CBT and a combination of both, in which the group with cognitive-behavioral intervention (n = 15) showed a significantly better treatment result compared to the group without cognitive-behavioral intervention (n = 14). Overall, behavioral therapy was already considered as “useful” at that time [19]. A non-randomized intervention study with 26 musicians from 2005 showed a positive effect on heart rate and superiority of situation-dependent use of propranolol compared to progressive muscle relaxation (PMR), but with a clear improvement in psychological well-being through PMR before and after a musical performance [7]. Overall, the study situation on the use of relaxation techniques such as PMR for the therapy of MPA is currently extremely limited (e.g. music-assisted PMR in pianists [20]). Moreover, there exist some recent studies investigating different approaches on therapy and coping strategies of MPA using acceptance and commitment therapy but also expressive writing [21–25]. Some studies have an explorative character with a single-subject design [22].

Considering all of the above, this study aims to close the existing gap by investigating therapeutic effects of cognitive-behavioral exposure therapy using virtual reality compared to a relaxation training. Besides psychological measures, physiological measures such as parameters for sympathetic activity measurable in the peripheral blood and cardiovascular reactivity will be considered as therapeutic outcomes.

With regard to benefits and harms, in the best case, the therapeutic interventions lead to an immediate and short-term reduction of music performance anxiety and could also provide an individual coping tool to increase self-efficacy in performance situations. Direct harms of the therapies are not to be expected. Of course, VR exposure may lead to experiencing anxiety and therefore to an increase in heart rate and blood pressure. For this reason, participants with a history of heart disease are not included in the study. Furthermore, the VR could lead to a simulation sickness in form of transient dizziness. In the case of PMR, there may be short-term muscle pain in some parts of the body. If complaints or disorders of the cervical spine are known ahead to the PMR intervention, the cervical muscle group is being omitted. Overall, these potential benefits could help promote participant retention and to motivate participants to complete follow-up.

### The application of virtual reality in psychiatry and psychotherapy and MPA

According to the German S3 guidelines for anxiety disorders, the therapy recommendation with the highest level of evidence is CBT, which includes exposure accompanied by a therapist [26]. Exposure therapy can be carried out in vivo and in a virtual reality (VR) setting [26]. Despite established therapy methods, only about a third of those affected receive therapy specifically designed for anxiety disorder (e.g. social phobia patients [27]).

In the case of virtual reality exposure therapy (VRET), the exposure takes place in a virtual space, which the subject can explore using special VR glasses. This type of exposure for anxiety disorders has already been tested in several large, controlled, randomized studies (for review in social anxiety disorders see [28]). It has been shown that VRET can not only trigger specific anxiety symptoms in patients with social phobia [29], but also reduce them just as effectively in the VR setting as exposure in vivo [30, 31]. VRET offers several advantages for both the therapist and the patient. The intensity of the feared situation can be tailored to the patient and the exposure session can be carried out directly in the treatment room [30]. This is especially the case for musicians, since a fear-triggering musical performance setting (with a jury or audience) is not always available and even more impeded in pandemic times. Furthermore, the threshold for seeking psychological help can also be lowered by using VRET [30].

In a case study in 2004, Orman first integrated a behavioral exposure approach with VR for music anxiety [32]. Some additional therapy studies [32–36] are using a VRET on MPA, although only small samples (n = 1–17) were examined. These five exploratory studies investigated the application of VR by musicians. In three studies, VR was used in terms of *exposure training* [32, 34, 35] and in two studies in terms of *simulation training* [33, 36]. However, musicians suffering from MPA in terms of a psychiatric disorder were not included. With regard to a psychometric assessments of anxiety only two studies used questionnaires such as the STAI-S and STAI-T [34]. In summary, Matei & Ginsborg [37] criticize the methodological weaknesses of many studies with too short study periods, small sample sizes, and a lack of controls. Although, larger and randomized clinical trials are still missing, all of these studies highlight the benefits of VR with far-reaching implications in simulation and performance training of musicians and call for further studies. In addition, Burin et al. [38] emphasize a “great need for preventive strategies and behavioral, educational, and pharmacological interventions”.

### Study aims and hypotheses

The study aims to evaluate the effectiveness of VRET for the treatment of MPA compared to an active control group receiving Jacobson’s PMR. The study sample comprises professional and semi-professional musicians and music students. The primary outcomes are change of MPA and heart rate variability. In addition, changes in stress parameters such as the cortisol response as well as immunological parameters, neuropeptides and epigenetic markers before and after a musical performance situation in VR will be examined.

The study addresses the following main questions: How effective is exposure therapy for MPA assessed by the reduction of subjective anxiety symptoms and objective cardiovascular parameters? We hypothesize that VRET will exert a significant and lasting reduction of subjective MPA symptoms at T1 (post/ approximately one week after the treatment) and at T2 (follow up/ six months after the treatment) compared to T0 (pre/ before the treatment). Also, we expect this reduction of MPA symptoms, at T1 and T2 compared to T0, to be significantly stronger for the experimental group receiving the exposure training compared to the control group receiving PMR. We further hypothesize that the postulated reduction of MPA in the experimental group goes along with a significantly higher HRV representing less cardiac stress during the musical performance situation in VR at T1 and T2 compared to T0. To conclude, the focus is primarily on the effectiveness of VRET compared to PMR.

## Methods and design

### Sample recruitment and selection criteria

Professional as well as semi-professional musicians and music students with different majors (e.g., artistic/ artistic-pedagogical/ pedagogical with an artistic focus), aged between 18 and 60 years and suffering from MPA, are included in the study. Further inclusion criteria are sufficient knowledge of the German language and compliance with Covid-regulations for local study appointments. Concerning the presence of a performance-disruptive MPA only musicians are included reporting a moderate or severe level of psychological distress (≥ 5 out of 10) and/or impaired functioning due to their fear of musical performance situations. This is assured by asking the subjects to rate on a scale ranging from 0 “not at all” to 10 “maximum possible” (1) how much they suffer due to their fear of musical performance situations and (2) how much they feel impaired in their functioning (i.e. every day or musical performance) due to their fear of musical performance situations. The musicians are included when rating at least one of the questions with at least 5 out of 10. Exclusion criteria comprise current or recent (< 6 months ago) psychotherapy because of the MPA and severe comorbid psychiatric or physical disorders, possessing a contraindication for exposure therapy (e.g. epilepsy, serious disorders of the cardiovascular system, psychotic disorders, borderline personality disorder, substance dependence or acute suicidality). Further exclusion criteria are concerning the characteristics and design of the study (e.g. small VR-cabin, assessment of blood samples, standing first-person view of the VR videos). Therefore, the subjects have to assure they are not suffering from severe claustrophobia or blood-injection-injury type phobia and can sing or play their instrument blindfolded and standing in the VR-cabin. For this reason and also because of the comparability of the HRV assessment, musicians with larger instruments and playing in a sitting position such as drummer, pianists, harpists and cellists cannot take part in the study. An overview of all inclusion and exclusion criteria can be found in Table 1. The subjects are recruited via local advertisements, flyers, radio announcements, and mailing lists of different musical universities, orchestras, music schools and theater groups in Bavaria.

**Table 1:** Overview of the selection criteria for sample recruitment

Selection Criteria	Description
**Inclusion Criteria**	Age between 18 to 60 years
	Anxiety and/or avoidance of musical performance situations (MPA)
	Moderate level (≥ 5/10) of physiological distress and/ or impaired functioning due to MPA
	Sufficient knowledge of the German language
	Compliance with the Covid-19-regulations for local study appointments
	Written informed consent
**Exclusion Criteria**	Physiological contraindications for an exposure therapy (i.e. epilepsy, serious disorder of the cardiovascular system)
	Psychological contraindications for an exposure therapy (i.e. substance dependence, psychotic disorder, acute suicidality)
	Severe Claustrophobia
	Severe Blood-injection-injury type phobia
	Use of sedative medication or tranquilizers (i.e. beta-blockers) for study appointments
	Instrument is not portable, too big for the VR-cabin, or not playable while standing
	Not able to play instrument blindfold
	Current or recent (< 6 months ago) psychotherapy due to the MPA

### Study design

The prospective, randomized trial has a pre-post design and includes a follow-up period of 6 months (see Fig. 1). Musicians (N = 46) with MPA will be examined before and after therapy.

At a first screening appointment (S), inclusion and exclusion criteria will be checked. The study information will be provided and written informed consent will be obtained. The study was approved by the ethics committee of the University of Würzburg (No. 194/21-sc). The project is in line with the Helsinki Declaration (as of October 2013, Fortaleza, Brazil) and Good Clinical Practice (GCP). Eligible musicians will be stratified by sex (male vs. female) and then randomized in two therapy arms, i.e. VRET or PMR. The randomization is carried out at the end of the screening appointment (S) by the investigators (DB or KW), who assign the musicians in chronological order to a previously defined random order of treatment conditions that has been generated by a computer. The subjects know from beginning about the random assignment of treatment conditions and they agree with it by filling the informed consent. They are told about their assigned intervention by the investigator (DB or KW) at the end of the pre- treatment assessment (T0) (single-blind randomization). Thereafter, screening questions concerning onset, form, and severity of MPA based on the criteria for specific phobia according to ICD 10 (F40.2) and social anxiety disorder according to DSM V (300.23, F40.10) will be addressed. A clinical interview for diagnosing mental disorders (Mini-DIPS-OA, [39, 40]) is conducted by a professional (DB and KW) to properly assess psychiatric comorbidities. Furthermore, information on the family history for affective disorders and cardiovascular diseases as well as current medication will be collected by questionnaires.

For each treatment condition (VRET vs. PMR) four individual therapy sessions within two weeks will take place on site at the University Hospital in Würzburg. Subjective as well as behavioral data will be collected at T0, T1 and T2, to describe the severity of MPA (see 2.5). For the assessment of the behavioral and physiological data a behavioral assessment test (BAT) in VR is conducted, during which the participant performs a musical audition (see 2.5.1).

In general, participants can discontinue or cancel the participation of the study at any time without giving reasons. In case of a high depressive or anxiety symptom burden in terms of worsening disease or even adverse events up to suicidal crisis, a psychiatric consultation will inform participants about alternative treatments, but this is not being realized as part of the study. Throughout the course of the therapeutic intervention modifying allocated interventions for a given trial participant are not provided (e.g. participant request, or improving/worsening disease).

This study was registered on clinicaltrials.gov. (ClinicalTrials.gov Number: NCT05735860; Protocol version of 02/09/2023 with ID: 194/21-sc)

### VRET

At the end of the pre assessment (T0) in the CAVE (computer assisted virtual environment) the subjects are given a psychoeducational manual in preparation for the VRET. They are instructed to read the manual at home and to complete short written exercises included in the manual. Contents of the manual are extracted from standardized CBT manuals and include the function and components of fear as well as their interplay (vicious circle of fear), information about MPA in discrimination to non-pathological stage fright and its treatment due to exposure therapy. Exercises concerning individual components of MPA, individual safety- and avoidance behaviors, and an individual *anxiety hierarchy* of the VR scenarios (see 2.3.1) were included in the manual to deepen the understanding of important key contents and in preparation for the exposure therapy. Before and after every VRET session, the study participants are given a questionnaire to assess important factors relating to the training progress and potential changes of the MPA due to the exposure training e.g. the adherence to the therapy rational. Furthermore, blood pressure and heart frequency are being measured in every training session, directly before and after the first exposure.

The first training session (VRET 1) starts with discussing the contents of the manual with the patients to make sure they understand the key points and mechanisms of behavioral exposure therapy. Sessions two to four (VRET 2–4) start with discussing the practice experience at home since the last session, reinforcing therapy compliant behavior and supporting the patient in case of difficulties with the exposure concept. The VR scenario for the exposure training is selected in every training session (VRET 1–4) and with every patient individually using the anxiety hierarchy. The selection criteria for the first scenario in VRET 1 is a moderate level of fear (approx. 60), which is considered high enough to elicit a fear reaction as suggested by Neudeck [41] but with the option for increasing the challenge for the next sessions (VRET 2–4). Individual concerns or preferences of the musicians regarding the scenario are considered. In preparation for the exposure, the expected physiological, cognitive, and behavioral reaction will be explored with the patients. Furthermore, they are asked to give fear ratings on a scale from 0 “no fear at all” to 100 “maximum fear” before (anticipatory and maximal expected fear), meanwhile (fear during the waiting phase) and after (maximal fear meanwhile the performance, fear after the performance) the exposure. After completing the scenario, the exposure experience is discussed with the patients focusing on important learning experiences (cognitive, behavioral, and physiological) for unlearning their fear of musical performance situations and on the proper execution of the exposure training (omitting safety or avoidance behaviors). If required, therapeutic support is given and instructions for further exposure scenarios are being discussed with the patients. The patients are exposed again to the scenario until either their fear of and during the performance situation has reduced to a minimum or until they have drawn an important learning experience regarding their fear and feel confident to proceed to another (more feared) scenario. Therefore, the exposure sessions accompanied by a therapist are aiming at (1) a between-session habituation/ reduction of the fear or (2) changes in the cognitive fear structure in the process of unlearning fear of musical performance situations. At the end of every training session (VRET 1–4) the musicians are instructed to practice similar performance situations in real life at home. Therefore a worksheet is given to them to facilitate the implementation. In the last session (VRET 4) positive learning experiences within the scope of the VRET are summarized together with the subject and a small theoretical input regarding relapse prevention and the handling of future performance situations is given.

The virtual environment is generated using the programs *GoProVR Player Version 3.0* and *Steam VR Version 1.20* and displayed via a *HTC Vive head-mounted display*. The VR scenario creates the exposure using 360° video sequences. Maximum duration of each session is one hour.

#### Description of 360° videos

**Fig. 2:**
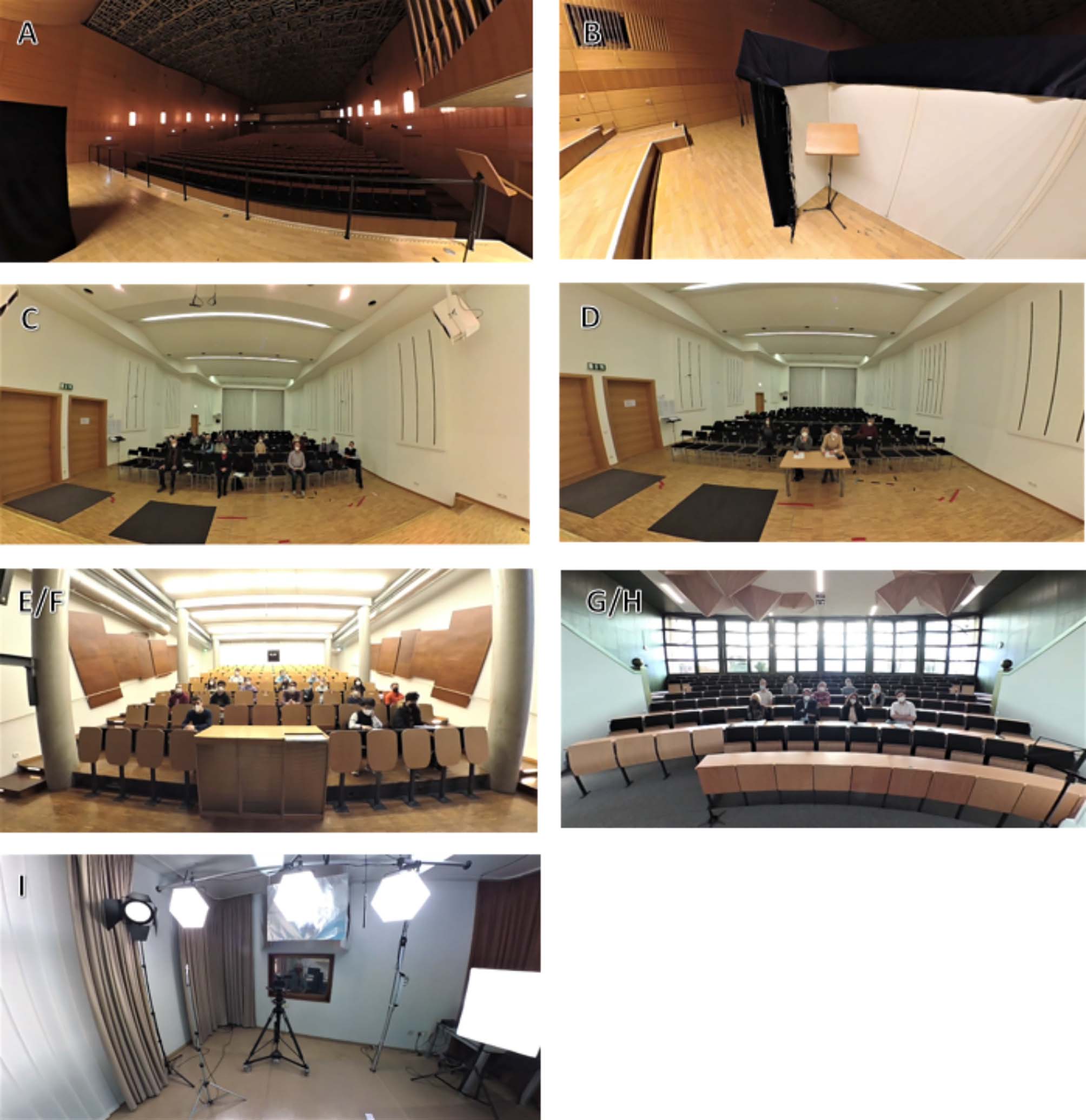
Pictures of the different 360° scenarios (A-I) from the perspective of the musician

Since the VR scenarios will be selected individually for every patient, an overview of the 360° videos is presented in Table 2 and in Fig. 2. The videos were filmed at locations of the University of Music in Würzburg and at the Center for Mental Health at the University Hospital in Würzburg. All participants seen in the videos gave their written informed consent.

Each video starts with a short 1.5-minute waiting phase in a backstage waiting room, where the participant can accommodate to the VR and prepare for the performance situation. The subjective fear rating is assessed verbally using a subjective units of distress (SUD) scale (0-100) before and after the feared situation as well as during the waiting phase. Scenario A consists of a professional jury (one man, two women) sitting in the audience of a large concert hall. The participant is located on a large stage at a distance of 4–5 m from the jury. The participant is asked by the jury to introduce him-/herself and the two pieces selected for the audition and then start to perform. Scenario B takes place in the same concert hall and has the order of sequence as scenario A, but differs in that the participant is performing behind a folding screen/paravent (similar to real audition situations) and thus unable to see the jury or the audience, and is interrupted by the jury while playing. Hence, scenario B has a total duration of 6.5 min (incl. the waiting phase). In scenario C, participants have to perform in a constrained area, i.e. on a small stage in a small concert hall in front of a professional jury (one man, three women). Two jurors (women) are sitting at a table in front of the first row in close distance to the musician. Similar to the procedure of the audition in scenarios A and B, after personal and musical introduction, the participant start to perform. After 2.5 min of performance, the participant is interrupted by the jury and asked to leave the stage. During the performance, the jurors take notes, carefully observe the participant and initiate eye contact amongst each other. Scenario D shows a concert situation in front of an audience consisting of 21 middle-aged people. It takes place in the same small concert hall as scenario C. When the participant enters the stage, the audience starts to clap hands. After ending the applause, the audience attentively listens to the performance of the musician. The scenario ends with the audience clapping hands enthusiastically after the musician finished the performance. Scenario E takes place in a classroom in front of an audience consisting of 17 music students displaying bored and distractive behaviors like talking to their neighbors, moving and changing seating positions, checking their phones, coming late to class, or leaving early. The Scenario has a duration of 6.5 min (incl. waiting phase). Scenario F takes place in the same classroom with the same audience of music students. In contrast to scenario E, the students in scenario F are listening attentively to the performance of the musician. The scenario ends with applause after the musician finished the performance. In Scenario G, the musician has to perform in front of the conductors and some ensemble members (five men, four women) of their (fictitious) favorite ensemble, which they have applied for. They are standing in close distance to the ensemble and are being asked by the ensemble director to present two pieces successively. Scenario H is similar to scenario G with the difference that another man is talking and that the subjects are being asked to explain their motivation of applying for the ensemble, similar to a job interview. The time for playing/ singing until getting interrupted by the jury is longer in scenario G (4 min per piece) than in Scenario H (3 min per piece). Scenario I takes place in a video studio presenting a live recording for a television broadcast. After entering the video studio, the musician stands in front of a green screen illuminated by floodlights. The musician is filmed and recorded by a camera and a microphone right in front of them. The musician can see a sound engineer through a small window, who is giving them instructions during the live recording. The total duration including waiting phase is 6.75 min.

**Table 2:** Overview of the 360° videos used for the VRET

Scenario	Description
A	Audition in front of a professional jury in a big concert hall
B	Audition behind a folding screen in a big concert hall
C	Audition in front of a professional jury in a smaller concert room
D	Concert in front of an elderly audience in a smaller concert room
E/F	Audition in front of a class of music students
G/H	Audition in front of the conductors and ensemble members
I	Live-recording for a television broadcast in a recording studio

### PMR

The content and procedure of the PMR training is aimed as equal as possible to the content and procedure of the VRET. The control group is given a psychoeducational manual in preparation for the PMR training sessions at the end of the first assessment in the CAVE (T0, Pre). They are instructed to read the manual at home and to complete small written exercises included in the manual. Contents of the PMR manual resemble contents of the VRET manual (e.g. function and components of fear, vicious circle of fear, information about MPA in discrimination to stage fright) adjusted to the different therapy method (e.g. treatment of MPA due to PMR). The use of PMR to reduce MPA is explained in the manual in terms of an anxiety management training. Psychoeducational contents specific to the VRET such as individual safety and avoidance behaviors, learning and unlearning anxiety are excluded from the PMR manual and replaced by PMR specific contents (e.g. the relaxation response, classical conditioning). Same as for the VRET the participants of the PMR training are given a questionnaire to assess important factors relating to the training progress and potential changes of MPA due to the relaxation training before and after every training session. Furthermore, blood pressure and heart frequency are being measured in every training session, directly before and after the PMR.

The content of the PMR manual is discussed with the patients in the first training session (PMR 1) and the patients have the opportunity to ask questions. They are introduced into the right execution of the PMR by explaining and practicing the correct way of straining and tensing the muscles together with the participant (PMR 1). Sessions two to four (PMR 2–4) are started with discussing the practice experience at home since the last session, reinforcing therapy compliant behavior and supporting the patient in case of difficulties with the therapeutic method. After that the musician is instructed to take a comfortable sitting position (e.g. take off shoes and glasses, unfasten waist belt, close eyes) and the PMR instruction is read out loud to the participant (PMR 1–4). The instruction used is a version by Bernstein and Borkovec according to the concept of progressive muscle relaxation in 16 steps by Jacobsen ([42], p. 56–58) with the three respective phases *focusing-tension-relaxation*. After having executed the 16 steps of tensing and relaxing certain groups of muscles, a one- to two-minute body scan is added. The PMR ends with a retraction phase instructing the participant to move, stretch and come back to the here and now. At the end of every session (PMR 1–4), the relaxation experience is discussed with the subject and if needed therapeutic support is given. The patients are instructed to practice the PMR at home to channel automation and to use the PMR for performance situations in case of beginning symptoms of anxiety. Same as for the VRET, a worksheet for documenting training sessions and experiences at home is given to the participants. Furthermore, the participants of the PMR group receive two audio files with the spoken PMR-instructions (female and male voice) to practice at home. In the last session (PMR 4) positive experiences within the scope of the PMR training are summarized and a short psychoeducational input regarding relapse prevention is given to the musicians.

### Assessments: before and after therapy, and at follow-up

In the course of the study different behavioral, neurobiological, physiological, and psychological assessments (see Table 3) are conducted at the different points of measurement (S, T0, VRET 1–4/ PMR 1–4, T1, T2). To record the fear reaction of the participants, various parameters of the emotion fear are measured. In addition to basic psychometry and SUDs, the physiological reaction (heart rate, blood pressure), and the endocrine reaction (cortisol in blood and saliva) are assessed. Molecular influencing factors are determined in the blood and therapy-related dynamics as well as exposure-related changes of the (epi-) genetic markers, the proteome, and metabolome are examined. Primary and secondary outcome parameters are described in detail in further chapters (see 2.6 and 2.7).

**Table 3:** Overview of assessments arranged to the type of measurement

Assessment	Screening (S)	Pre (T0)	VRET 1–4/ PMR 1–4	Post (T1)	Follow-up (T2)
Behavioral					
BAT		X		X	X
**Neurobiological**					
Blood sampling		X		X	X
Saliva sampling		X		X	X
**Physiological**					
HRV		X		X	X
Heart rate		X	X	X	X
Blood pressure (RR)		X	X	X	X
**Clinical and psychological**					
Diagnostic criteria for MPA as social phobia (DSM-5) and as specific phobia (ICD-10)	X				
Mini DIPS	X				
Bühnenangstfragebogen (BAF)		X		X	X
Kenny Music Performance Anxiety Inventory (K-MPAI)		X		X	X
Fragebogen zum Sicherheits- und Vermeidungsverhalten bei Musikerinnen und Musikern		X		X	X
Social Interaction Anxiety Scale (SIAS)		X		X	X
Social Phobia Scale (SPS)		X		X	X
Beck Depression Inventory (BDI-II)		X		X	X
Beck Anxiety Inventory (BAI)		X		X	X
Anxiety Sensitivity Index (ASI-3)		X			
State Trait Anxiety Inventory (STAI-Trait)		X			
Childhood Trauma Questionnaire (CTQ)		X			
Immersive Tendencies Questionnaire (ITQ)		X			
Uncertainity Tolerance Scale (UGTS)		X			
Positive and Negative Affect Schedule (PANAS)		X		X	X
Questionnaire CAVE Pre_before BAT		X			
Questionnaire CAVE Pre_after BAT		X			
Questionnaire CAVE Post_before BAT				X	
Questionnaire CAVE Post_after BAT				X	
Questionnaire CAVE Follow-up_before BAT					X
Questionnaire CAVE Follow-up_after BAT					X
SUDs		X		X	X
Questionnaire_before VRET/PMR			X		
Questionnaire_after VRET/PMR			X		

Questionnaires: BAF [43], K-MPAI [44], Fragebogen zum Sicherheits- und Vermeidungsverhalten bei Musikerinnen und Musikern ([45], p. 207), SIAS [46, 47], SPS [47, 48], BDI-II [49], BAI [50, 51], ASI-3 [52], STAI-Trait [53, 54], CTQ [55], ITQ [56], UGTS [57], PANAS [58] and further questionnaires being assessed before and after each BAT and therapy training session.

#### Behavioral assessment in the CAVE

In order to assess MPA in a condition as real as possible on the different levels (subjective and physiological), a BAT is performed in the CAVE (software from VTplus GmbH, Institute of Psychology I at the University of Würzburg). The CAVE system is a room sized 4 × 3 × 3 m, in which a virtual environment is projected onto the four walls and floor via stereoscopic images. The stereoscopic impression is created by passive interference filtering glasses worn by the subjects [59]. The test person can move around freely with his/her instrument without any problems. During this behavioral test, the participants are instructed to present two different pieces of music over approximately 10 min playtime. In that time of the behavioral test the HRV will be measured simultaneously, using appropriate sensors and electrodes (*EcgMove 4* by Movisens®). Additionally, subjective fear ratings (SUDs) will be assessed.

After finishing the preparations for the BAT (e.g. attaching the microphone, head tracker, and electrodes for measuring the heart rate), blood pressure and heart rate are measured. The musicians are instructed to behave and interact with the jury (e.g. respond to questions) as if it was a real audition. Also, they are briefed about the subjective fear ratings ranging from 0 “no fear at all” to 100 “maximum fear” that are assessed throughout the BAT over a microphone. When entering the CAVE, a physiological baseline for the HRV is recorded. Therefore the participants have to sit and to stand respectively one minute in a neutral CAVE (white walls) while doing nothing. After measuring the baseline, the subjects are asked to rate their anticipatory and maximum expected fear of the audition. Subsequently, they are told that the audition will start now and the CAVE shifts to display a waiting room in which the musicians find themselves waiting in front of the door of a big concert hall. To help the participants immersing into the situation, short, standardized instructions are read at the beginning of the waiting phase and when entering the stage. At the beginning and at the end of the waiting phase, the participants are asked to rate their actual fear (0-100). Thereafter, participants “enter the stage” by shifting the CAVE to display a concert hall with other musicians waiting in the background and people sitting in the audience. The musicians are instructed to wait for another minute until the jury will arrive, and to feel free to look around and prepare for their performance. Also they are asked again to rate their current fear (0-100). Finally the jury, consisting of two men and two women, pops up in the first row of the audience and another fear-rating (0-100) is assessed. When the jury appears they seem busy (e.g. looking down at their notes or around). After 30 s all the jury members lay back and start to look up at the participant and a male juror begins to greet the participant. He asks the musician to introduce her-/himself and the first piece he/she is going to perform. When the musician has finished answering, the juror reacts confirming the choice of the piece and asks the musician to start playing/ singing when ready. After three minutes, the participants are being interrupted by the jury even though they have not finished their first piece yet. Another fear rating is assessed asking for the maximum fear while playing (0-100) and the actual fear after having finished the first piece (0-100). Finally, a female jury member asks the musician for the second piece that the musician is going to present and prompts the participant to start the performance. Following the same procedure as for the first piece, the musician is being interrupted by the jury at latest after having played three minutes, which is followed by the fear ratings to assess the maximum fear while playing (0-100) and the actual fear after having finished the second piece (0-100). Thereafter, the female jury member says goodbye and asks the participants to wait outside. The audition is ended and the BAT ends with again recording a physiological baseline for the HRV in a neutral CAVE (e.g. white walls), same as at the beginning. After recording the baseline, the participants are asked for the last time to rate their actual fear (0-100) and are then asked to leave the CAVE. Outside the CAVE, blood pressure and heart rate are measured again and microphone, head tracker and electrodes are being removed.

In addition, during the BAT, the musical performance is recorded by a recorder (Zoom H4n®) which is attached to a microphone stand in front of the musician.

The total duration of the CAVE examination is estimated to last approximately 120 min of which 50 min are estimated for the BAT, its preparations and debriefing (e.g. attaching the microphone, head tracker and electrodes for measuring the heart rate, recording the physiological baseline and measuring heart rate and blood pressure before and after the BAT), 40 min to fill out the questionnaires, and 30 min to take blood and saliva samples in the beginning and at the end of the examination.

Concerning the pieces of music, the participants are told two weeks in advance to present a slow and a virtuosic piece of music of moderate to high degree of difficulty, similar to a real audition. Participants have to present the same two pieces of music at all time points (T0 up to T2). The follow-up (T2) after six months also takes place at the CAVE and an appointment is made with the participants directly after the therapy respectively the assessment in the CAVE at T1. By doing so, we hope to promote participant retention and motivate them to complete follow-up.

#### Blood samples

At the CAVE examination (BAT, T0/T1/T2) blood samples include 2 × 1.6 ml EDTA for (epi-)genetics, 1 × 1.6 ml EDTA for blood count, 2 × 9.0 ml EDTA for proteins, 2 × 9.0 ml serum for metabolome and endocrine analysis as well as saliva samples of 2 × 1.1 +/- 0.3ml. In total, this amounts to 3 × 37.8 ml blood samples as part of the CAVE examination.

### Primary outcome parameters

The primary outcome parameters are therapy effects on MPA measured by the Bühnenangstfragebogen (BAF) as well as changes in cardiac reactivity using HRV. Therapy response criteria for MPA are defined by a decrease of at least 50% of the BAF total score compared to T0. Remission ist defined by no psychological distress or impaired functioning due to the symptoms. Regarding the cardiac reactivity, HRV is not yet sufficiently established as a biomarker for music performance and there is no reliably data in this area so far which is why we evaluate this data exploratively. However, in case of a successful reduction of MPA we expect a significant higher HRV during the BAT after therapy compared to before therapy.

#### Psychometric assessments for MPA

Specific questionnaires on performance anxiety have already been established since the 1990 [4, 60]. As dimensional measures of MPA the German *Bühnenangstfragebogen* (BAF) [43] and a german translation of the revised Kenny Music Performance Anxiety Inventory (K-MPAI-R, 42) are assessed to measure subjective symptoms of MPA. Since the K-MPAI (K-MPAI-R, 42) has not been validated yet and is considered less sensitive to detect chances in MPA over time than the BAF [2, 45], we only consider the BAF [43] as primary outcome measure in this study.

The BAF [43] is a validated german translation of the Performance Anxiety Questionnaire by Cox and Kenardy [60] and consists of 20 Items assessing MPA separately for solo and ensemble performances. The items are measuring cognitive and bodily symptoms of MPA and have to be rated by their frequency of occurrence during performance situations (“How often do you experience those thoughts or feelings meanwhile a musical performance situation?”) on a five-point scale ranging from 1 “never” to 5 “always”.

BAF scores are assessed before the BAT at pre- (T0), post- (T1) and after six month follow-up-treatment (T2) to detect changes in MPA symptoms over time. We hypothesize that musicians of the experimental group show significantly lower overall score measured by the BAF [43] at T1 and at T2 compared to T0 representing a successful VRET with lasting effects. Furthermore, we expect this reduction of the BAF [43] overall scores to be significantly stronger the experimental group (VRET) than for the control group (PMR).

#### Primary outcome: heart rate variability

Heart rate variability (HRV) describes the variability in the time intervals between consecutive heartbeats. Since our cardiovascular system has to adapt constantly to changing conditions to adjust to physical and psychological challenges, the oscillations of a healthy heart are complex and constantly changing [61]. Measuring HRV provides a non-invasive method to assess the cardiac autonomic modulation. Since its easy application and tolerability to the proband, HRV is widely used and well established in different scientific fields, not only applied in humans but also animal studies. Today, common HRV devices record a one-channel electrocardiography, from which the heart rate and the HRV can be derived, but are also capable to measure additional parameters such as body temperature, acceleration, and air pressure.

There are many different approaches to calculate HRV. A detailed description about HRV metrics and norms, and the different parameters is given by Ginsberg and Shaffer [61]. Giving a brief summary, HRV can be described using *time-domain bound approaches* like the standard deviation of normal-to-normal intervals (SDNN) and the root mean square of successive differences (RMSSD), *frequency-domain bound approaches* like high frequency power (HF) and low frequency power (LF), and *non-linear approaches*. All parameters are rooted in the variability observed between consecutive R waves (RR-intervals), which is why they are highly correlated. Till this day, the superiority of any parameter across settings has not been demonstrated, even though it has been shown that some parameters represent different aspects better than others [61–64]. Selecting the recommended measure and interpreting the data, context factors and subject variables have to be considered e.g. method and length of recording (24 h, short-term (~ 5 min), ultra-short-term (< 5 min)), sampling frequency, removal of artifacts, respiration, position, movement, tasks and demand characteristics, age, sex, heart rate and health status [61].

In the scientific discourse, some authors emphasize the value of HRV even as a „biomarker“ [65, 66] respectively as a „psychophysiological marker for physical and mental health“ [64, 67]. A current meta-analysis on HRV in patients with anxiety disorders found that especially resting-state HRV in patients with anxiety disorders, in general, was significantly lower than in healthy controls with a low to a medium effect size of Hedges’ g 0.39 reflecting a „robust feature of anxiety disorders“ ([68], p. 9).

With regard to music performance, HRV has already been investigated in some studies and described as a correlate of fear in the context of performance anxiety [8, 14, 69] but has not been established as a Biomarker. Just a few studies suppose a correlation between autonomic cardiac modulation and the flow experience during music performance [70, 71]. Concerning therapeutic interventions, a randomized-controlled trial investigated a single-session biofeedback training on MPA and HRV with positive effects on HRV of a slow breathing group in comparison to controls [67]. Two studies on anxiety levels during music performance using VR already investigated HRV, although the sample size was very small (30, n = 5, 34). In particular, Williamon et al. used HRV to calculate the LF/HF ratio with a subgroup of violinists (n = 7) and found significant differences emerged in physiological responses for a pre-performance period (real audition vs. simulated) which interestingly did not correspond to higher anxiety scores assessed with the STAI-Y1 [72]. Referring to the impact of context factors as mentioned above the type of instrument (wind, strings, singing, etc.) and its position of playing could play a decisive role in HRV analysis which has not yet been investigated sufficiently in musicians.

In our study, we use primarily a biobehavioral approach [73] investigating the following parameters of HRV: heart frequency [bpm], SDNN I [ms], RMSSD [ms], pNN50 I [%], LF [ms2], HF [ms2] and LF/HF-Ratio. The HRV is assessed meanwhile the BAT and we expect a higher HRV to represent less cardiac stress. Therefore, we assume that a successful reduction of MPA goes along with a significant higher HRV in the BAT at T1 and T2 compared to T0.

### Secondary outcome measures

Blood-based parameters are easy to access in patients and therefore could have an immense use in diagnostic and predictive ways within the personalized medicine approach. In the present study, we focus on stress-related endocrine measurements, neuropeptides and DNA-methylation as measurable parameters in the peripheral blood to explore (1) their utility as a proxy for the fear-related phenotype, (2) potential predictive use for therapy response and (3) the dynamic changes within the therapy course.

Stress is one of the important factors contributing to the development and relapse of various anxiety disorders. In a recent meta-analysis, cumulative HPA-axis parameters and autonomous nervous system markers were associated with anxiety arousal and fear severity [74]. Given the first reports on CBT-related attenuation of HPA system activation in anxiety disorders, we hypothesize that successful VR exposure treatment leads to the reduction of stress elicited cortisol levels within the BAT to both time points, T1 and T2 and that this reduction is related to clinical response. Furthermore, we will analyze whether the magnitude of cortisol release during the T0 BAT influences the clinical response to T1 treatment effects in a predictive way. Lastly, we investigate, how cortisol levels in T0 and T1 can project on MPA symptoms 12 months after the treatment (T2).

Neuropeptides serve as messenger substances or as co-transmitters, i.e. with 5-HT, GABA or norepinephrine. A number of neuropeptides have been shown to modulate anxiety-related behavior in various preclinical models [75]. In the present study, we focus on neuropeptides with modulating effects on humoral and behavioral stress response, and myocardial as well as autonomic nerve system function (ANS), such as Atrial natriuretic Polypeptide (ANP) (Marazziti et al., 2021), neuropeptide Y (NPY) [76], oxytocin (OXT) [77], and the enzyme salivary Alpha-Amylase (AA) [78].

Genetic factors contribute to the aetiology of anxiety disorders (AD) with heritability estimates ranging around 20–40% for special phobias and social anxiety disorder [79]. Following this, environment acts as an important contributor to molecular processes which lead to the development of pathological anxiety states in both conditions [80]. In this context, epigenetic processes have been increasingly studied in AD as a possible link between environment and molecular function [81]. Epigenetic modifications describe gene regulatory processes without changing the original DNA, such as micro-RNA (miRNA), histone modifications and DNA-methylation (DNAm), the latter being the most studied epigenetic mechanism in psychiatry to date. An increasing number of epigenetic studies suggest that the dynamics of DNAm and subsequent gene regulatory changes modulate the effects of psychological therapies and could serve as a possible biomarker for short- and long-term therapy success [82]. In the present study we investigate mDNA of the candidate genes related to the endocrine stress reactivity (Glucocorticoid receptor gene (*NR3C1*), FK binding protein 5 (*FKBP5*), corticotropin releasing hormone receptor 1(*CRHR1*)), which is hypothesized to be modulated by successful exposure therapy. Furthermore, Norepinephrine Transporter (*NET*) and Oxytocin Receptor (*OXTR*) genes were selected for DNAm analysis due to their implication in the regulation of autonomous function and social anxiety.

Regarding cardiac stress by measuring heart frequency and blood pressure and regarding MPA by assessing SUDs during the BAT, a significant difference indicating less cardiac stress or lower ratings of MPA during the BAT between the time before and after therapy is expected.

Summarizing, an overview of all primary and secondary outcome parameters and its measures is given in Table 4.

**Table 4:** Overview of the constructs and measures

Construct	Measure
Primary Outcome	
MPA symptoms	Bühnenangstfragebogen (BAF)
Cardiac reactivity	Heart rate variability (HRV)
**Secondary Outcome**	
MPA during BAT	Subjetive units of discomfort (SUDs)
Social anxiety symptoms	Social Interaction Anxiety Scale (SIAS)
Social phobia symptoms	Social Phobia Scale (SPS)
Depression symptoms	Beck Depression Inventory (BDI-II)
Anxiety symptoms	Beck Anxiety Inventory (BAI)
Anxiety sensitivity	Anxiety Sensitivity Index (ASI-3)
Trait anxiety	State Trait Anxiety Inventory (STAI-Trait)
Childhood-history of abuse and neglect	Childhood Trauma Questionnaire (CTQ)
Immersive tendencies	Immersive Tendencies Questionnaire (ITQ)
Uncertainty Tolerance	Uncertainity Tolerance Scale (UGTS)
State affect	Positive and Negative Affect Schedule (PANAS)
Cardiac stress	Heart rate, Blood pressure
Psychophysiological stress response	Salivary Cortisol hormone
	Salivary Alpha-Amylase hormone Atrial natriuretic Polypeptide (ANP)
	Neuropeptide Y (NPY)
Psychosocial stress response	Oxytocin (OXT)
DNA-methylation in peripheral blood	Glucocorticoid Receptor (*NR3C1*), FK binding protein 5 (*FKBP5*), Corticotropin releasing hormone receptor 1 (*CRHR1*), Oxytocin Receptor (*OXTR*), Norepinephrin Transporte (*NET*)
Subjective MPA symptoms	Kenny Music Performance Anxiety Inventory (K-MPAI)
Use of MPA specific avoidance and safety behaviors	Fragebogen zum Sicherheits- und Vermeidungsverhalten bei Musikerinnen und Musikern

### Data collection, data processing, data confidentiality and statistical analysis

Study data will be collected and managed using REDCap electronic data capture tools hosted at University Hospital Würzburg [83, 84]. REDCap (Research Electronic Data Capture) is a secure, web-based software platform designed to support data capture for research studies.

During the study, the assignment of the participants to the collected data is carried out via a written assignment list, which is only accessible to the investigators. The collected data is stored in pseudonymized form. In case of a revocation, the data already collected will be deleted and, if desired, no longer processed. The data will be stored pseudonymously for a period of 10 years.

The primary parameter for the evaluation of the therapeutic effects of this study is the MPA questionnaire, with the expected superiority of VRET group compared to the relaxation training group regarding the decrease of the reported symptomatology immediately after the therapy.

For the primary analyses, an intention to treat (ITT) analysis will be performed for all included participants, except for those withdrawing consent during the study or not receiving at least one treatment. Missing values will be imputed using a last value carried forward approach. No interim analysis will be performed. The analysis is performed by ANCOVA with measurement repeat factor time (pre-post), the factor group (experimental-control), and the baseline values of the respective variable as covariates.

Regarding HRV, we analyze the heart rate (bpm) and RMSSD as an HRV indicator. Heart rate and RMSSD each serve as a model outcome in multivariate analyzes with multiple predictors (e.g. age, gender, instrument group).

#### Sample size calculation and number of VR sessions

There are only a few studies that have examined VR and in particular VRET for treating MPA [35, 37]. Samples sizes were very small ([32], case study, n = 5; [34], n = 17) and with regard to their methods extremely heterogeneous. Likewise, the study situation on the use of PMR for performance anxiety is extremely small (e.g. music-assisted PMR in pianists, [20]; PMR vs. propranolol, [7]. Corresponding meta-analyses are lacking.

A recent meta-analysis by Carl et al. [85] from 30 studies with 1057 patients examined the effectiveness of VR exposure for anxiety and anxiety-related disorders in general. Here, a large effect size was reported for the VRET, with a Hedges’ g of g = 0.88 compared to waiting list conditions (g = 0.88, SE = 0.10, 95% CI: 0.69–1.07). Compared to an active control condition, such as relaxation exercise including PMR, the effect size was g = 0.78 (SE = 0.27, 95% CI 0.25–1.31). This corresponds to an effect size of f = 0.39, which we used in GPower (version 3.1.9.7, alpha = 0.05, power = 0.95, r = 0, with 2 groups and 2 measurements (pre/post)) to determine the sample size for a significant interaction used in the between-group and time-point repeated measures ANOVA. The total sample size was calculated as n = 46. Taking into account a drop-out of around 10% during the study, 52 patients are defined as a sufficient total sample size for the planned examination of MPA.

Regarding the number of VR sessions mentioned in this meta-analysis [85] in the case of specific phobias, such as fear of performing, very good effects were achieved with 4 sessions. Even with just one intervention, there was an effect size of g = 0.88 with n = 77 participants [86]. In studies on social phobia, which defines complex symptoms with relevant limitations in several social situations in addition to performance anxiety, Harris et al. [87] still report a g = 0.91 with 4 sessions ([85], Table 2). The only VR study on performance anxiety listed in this meta-analysis conducted a total of 6 exposure sessions on music students with a Hedges’ g of g = 0.14. However, the examined music students did not suffer from clinically diagnosed performance anxiety, which reflects the weak evidence within this form of anxiety disorder as described earlier (n = 17; [34]). The authors of this meta-analysis state in summary that “there was no effect for the number of treatment sessions in either VRET versus control (p = 0.87) or in vivo comparisons (p = 0.67)“ [85, p. 34].

In addition, an even more recent meta-analysis (11 studies with 626 patients) examined the benefit of cognitive-behavioral therapy with the additional use of VR (Virtual Reality-Assisted Cognitive Behavioral Therapy, VRCBT) and was able to demonstrate positive effects of VRCBT in anxiety disorders under these conditions ([88], p. 7, SMD − 0.92), although calculations of effect size on the number of sessions were missing. The authors conclude that further research is needed to confirm the benefit of VRCBT with different subtypes of anxiety disorders (88, p. 8). In this context, MPA, a subentity of social anxiety, can be listed here.

In the light of this knowledge and taking into account that the majority of our participants in the study does not suffer from any general social phobia, but rather a very defined performance anxiety as mentioned above, 4 sessions for the sample size are considered as sufficient and we do not expect any loss of power.

## Discussion

### Questions on MPA, that can be answered

The study aims to investigate the effect of exposure treatment in musicians with performance anxiety compared to a relaxation technique on anxiety symptoms and corresponding cardiovascular parameters in musicians.

To our knowledge, this is the first randomized controlled study on MPA which will investigate different psychological AND physiological parameters in a therapeutical context using VR. Randomized controlled trials on MPA have been rare since the last 30 years [19, 67, 89] and have long been demanded within this field considering this neglected subentity of anxiety disorders and in particular the neglected application of virtual reality in MPA [1]. Therefore, therapeutic implications are diverse and the study is capable to answer the following questions.

First, regarding music performance anxiety itself, it remains unclear if a music performance on a “virtual stage” in an artificial environment is able to generate the same stress or anxiety level in musicians as in vivo. By using various psychometric assessments which has been chosen carefully and critically to measure anxiety and its covariables this study can answer this question solidly. Second, in addition, the study can also provide information about how cardiovascular reactivity and cardiac stress reflected by the heart rate variability and blood pressure will emerge in a virtual setting. As highlighted above, in the literature, there are just a few studies that address the association between performance anxiety and cardiovascular reactivity in musicians [7, 14, 15], but there are no studies in particular that assess the impact of MPA therapy on sympathetic tone and cardiovascular reactivity. Furthermore, physiological and epigenetic correlates of MPA (cortisol, neuropeptides, DNA-methylation) will be investigated and answer the question if and how strong there are measurable changes while performing in a VR environment and in relation to therapy response. Third, with regard to therapy, this study can answer the question if and how strong a reduction of anxiety but also a consecutive improvement of HRV with cardiovascular protective effects can be achieved. Fourth, the study can provide valuable information whether both treatment protocols might be useful as prophylactic intervention for musicians who experience distress without being yet affected by MPA.

In this context, follow-up studies, e.g. multi-center studies with the participation of different universities of music in Germany would be helpful to identify those young music students who have or might be at risk to develop MPA and to offer therapeutic strategies based on study results.

### Limitations

VR setup might be not efficient to evoke the psychological and physiological correlates of MPA, which are a prerequisite of successful exposure therapy. With regard to the external circumstances of the SARS-CoV2 pandemic and all their manifold implications the 360° VR exposure material has been created under considerable restrictions and hygiene regulations. By this time now, we do not know how and to which extend the face masks compromise the maximum effect of the virtual environment evoking a high anxiety. All actors shown in the VR videos are wearing face masks making it difficult to impossible for the probands to recognize and differentiate human facial expression with the head mounted display. Emotional expressions of different valences such as surprise, joy, astonishment, rejection, or rigidity which are essential for an interaction of the proband with the VR cannot be generated sufficiently. This could have a positive or negative impact on musician’s performance anxiety. On the one hand, some musicians could experience it as a relief and protective barrier, on the other hand it could be interpreted as a “lack of human feedback” from the audience. However, throughout the course of the pandemic people certainly have gotten used to face masks and have learned to practice more eye and body reading with their interaction partner. Indeed, participants included in the study report current high MPA levels evoked in the present pandemic situation suggesting that exposing the current real life condition in VR will lead to high anxiety levels. Furthermore, the number of spectators was limited due to the social restrictions and spatial distance among themselves within the room or concert hall. A crowded concert hall setting for example, triggering probably the highest anxiety levels for some musicians could not be realized. Only a small audience of 17 up to 21 people was possible to record. Furthermore, the nuances of an audition setting with the authority, rigidity or benevolence of the judges can be shown as just rudimentary. To summarize, it remains unclear at this moment how these technical and social factors are negatively assessed by the probands and to which extent the VR setting is being impaired in its effectiveness. Finally, this exposure-based VR therapeutic setting allows us to work with the musician individually, but not to set individual therapeutic priorities e.g. focus on cognitive, physiological or behavioral symptoms in a recessed way. Musicians might further benefit from additional interventions on an individual level, therefore, further studies should explore the effect of VR therapy *in combination* with these various methods in psychotherapy to increase the beneficial therapeutic outcome in patients with MPA.

### Impact on the definition of MPA

The diagnosis of MPA in participants of this study has been profoundly assessed by professionals based on a structured clinical interview and not just by self-rating questionnaire respectively self-assessment instruments as in past studies [4–6, 38, 60, 90]. This fact is essential to differentiate *music performance anxiety* properly from physiological *stage fright* which is being discussed further. In the literature, as outlined earlier, the term MPA is often used arbitrarily and synonymously with stage fright throughout the decades [4, 19] and with partially inconsistent definitions [34]. Fernholz et al. rightly state that a “consensus on its definition has not been reached yet” [1] which actually refers to a statement of Kenny in 2005 that “the terms [MPA or stage fright] are used interchangeably” [91]. These terms do not depict MPA sufficiently as a complex phenomenon with its cognitive, emotional, physical and behavioral components [37]. According to the international classification of diseases, it is defined as a specific phobia whereas in DSM 5 the entity is classified as social anxiety, subtype “performance only”. However, the term itself is considered problematic. In our point of view, MPA has to be separated from the physiological “straight fright” (German “Lampenfieber”) every musician knows very well which reflects a physiological state and positive level of attention and focus resulting in an increase of musical performance quality on stage without any pathological value analogous to the already mentioned *Yerkes Dodson’s law* as describes by Mumm et al. [2]. In contrast, MPA indeed has a pathological value for those affected especially the extent of individual impairment at different stages. As already mentioned, in terms of a psychiatric disorder it can be defined as a subgroup of social anxiety disorder and is characterized by the extent of the anxiety, the subjective level of suffering as an emotional burden as well as the functional impairment which inevitably leads to reduced artistic performance. As described above, research on this topic has been done for about 30 years with an exceeding heterogeneity concerning methodology and sample size of the studies which tightened the problem of the widespread definition ambiguity. In many studies, the terms music performance anxiety or stage fright are often used synonymously making it difficult for professionals to choose an evidence-based therapeutic intervention. This circumstance confirms our point of view that a discourse about this topic is not just an academic question for the therapeutic implications are essential for those who are searching for adequate therapeutic help and concerning the fact that just 15% of those affected musicians seek professional help [3]. Kenny has already drawn attention to the “lack of a clear definition of MPA” in 2010 which “hinders the development of appropriate treatments.” ([3], p. 433). Thus, in the last years, this fact has been recognized in the scientific discourse and a few studies address especially this issue. However, from our point of perspective, MPA is not defined properly in the context of a psychiatric disorder, and Fernholz et al. in particular request a consistent definition of MPA [3].

Therefore, we intend to provide a small but important difference in the definition and terminology: we propose the term *Music performance anxiety disorder* when using it to describe a pathological state of performance anxiety in contrast to *stage fright* as the “benign form” and positive arousal in the context of music performance on stage. This will help first, to provide adequate therapeutic interventions for those musicians and second, also to better communicate with patients *and* professionals in the field of psychiatry and psychotherapy. Moreover, in exactly this context, the objective measurements assessed in our study of two established therapeutic interventions (VRET and PMR) pre and post - psychometric and physiological parameters - will hopefully support this idea of a new terminology, speaking of *Music performance anxiety disorder* in terms of a new pathophysiological concept with concrete therapeutic implications for the musicians without interprofessional misunderstanding.

### Outlook

To conclude, music performance anxiety is a common but complex phenomenon among professional musicians but also semi-professional musicians which should no longer be neglected or trivialized by professionals in the field of music *and* medicine. The complexity of the disorder and also its cardiac impact on musicians should be considered properly in a therapeutic context. Indeed, cardiovascular risk factors such as hypertension, hypercholesterolemia, etc., increase inevitably with age and can have an unfavorable effect in the context of MPA especially when changeable risk factors in musicians are involved (nicotine abuse, lack of physical exercise and so forth). Given the fact that MPA as other anxiety disorders is accompanied by a prolonged and permanently increased sympathetic activation exposure therapy may help to decrease sympathetic activation and reduce anxiety symptoms which could in turn result in a cardiovascular protective effect.

15% of musicians with MPA seek professional help. This in turn means that many of those affected may be able to cope well with it on their own and find a good way to deal with performance anxiety in the course of their artistic career. The much-discussed argument of wanting to pathologize music-making and stage fright can be invalidated. Of course, finding a new definition and therapy for MPA is about helping musicians to get professional support quickly and to effectively enable affected musicians to better cope with the problem themselves.

Finally, VR-based exposure treatment can contribute in a multimodal therapeutic concept for musicians by (1) providing an important impetus to better help themselves and (2) to strengthen and increase their sense of self-efficacy in terms of a practical therapeutic tool in everyday life by transferring and applying it in vivo exposure. VRET could help to initiate an adequate therapeutic intervention in early stages with a short and limited number of VR sessions [4–6] to shorten the period of individual suffering and counteract a chronification process.

## Data Availability

Not applicable.

## List of abbreviations

ANCOVA: Analysis of covariance.
BAF: Bühnenangstfragebogen.
BAT: Behavioral assessment test.
BPM: Beats per minute.
CAVE: Computer assisted virtual environment.
CBT: Cognitive behavioral therapy.
DSM-5: Diagnostic and Statistical Manual of Mental Disorders (5th edition).
HRV: Heart rate variability.
ICD-10: International Classification of Diseases (10th revision).
ITT: Intentions to treat.
K-MPAI-R: Revised Kenny Music Performance Anxiety Inventory.
MPA: Music performance anxiety
NP: Neuropeptides
PMR: Progressive muscle relaxation. S: Screening
S: Screening
SUD: Subjective units of distress.
VR: Virtual reality.
VRBCT: Virtual reality-assisted cognitive behavioral therapy.
VRET: Virtual reality exposure therapy.
